# Obstructive sleep apnea screening performance of the STOP-BANG questionnaire and a home sleep apnea test device in atrial fibrillation ablation candidates

**DOI:** 10.1007/s10840-025-02131-7

**Published:** 2025-10-18

**Authors:** Jasper Vermeer, Maarten van den Broek, Tineke Vinck-de Greef, Hennie Janssen, Pauline van Hirtum, Sebastiaan Overeem, Lukas Dekker

**Affiliations:** 1 Department of Cardiology, Catharina Hospital Eindhoven, Michelangelolaan 2, Eindhoven, 5623 EJ the Netherlands; 2 Department of Electrical Engineering, Eindhoven University of Technology, Eindhoven, the Netherlands; 3 Sleep Medicine Center Kempenhaeghe, Heeze, the Netherlands

**Keywords:** Atrial fibrillation, Sleep apnea, STOP-BANG, Home sleep apnea test, Polysomnography

## Abstract

**Background:**

Obstructive sleep apnea (OSA) contributes to the onset and progression of atrial fibrillation (AF) and negatively affects AF ablation outcomes. OSA screening in AF patients is often conducted with the STOP-BANG questionnaire, although its validation is lacking. This study aims to evaluate the screening value of the STOP-BANG questionnaire and a home sleep apnea test (HSAT) device for OSA in patients referred for AF ablation.

**Methods:**

Patients referred for their first AF ablation and without prior OSA diagnosis underwent both the STOP-BANG questionnaire and a HSAT device based on peripheral arterial tonometry (PAT). Patients with a PAT-derived apnea-hypopnea index (pAHI) of 5 or more events per hour subsequently underwent clinical polysomnography (PSG). This PSG was used for definitive OSA diagnosis and to determine the diagnostic values of the STOP-BANG and HSAT.

**Results:**

Of 67 patients initially screened with the STOP-BANG and HSAT, 58 completed PSG after excluding those with pAHI < 5/hour or who declined further testing. Among these 58 patients, STOP-BANG (score ≥ 3) correctly screened 84% of cases, while HSAT was more accurate (97%, *P*-value < 0.002). Among the 67 initially screened patients, 56 (84%) received a new OSA diagnosis. Of these, 21 (38%) had mild OSA, 17 (30%) moderate OSA and 18 (32%) severe OSA.

**Conclusion:**

The high OSA prevalence highlights the importance of OSA screening in patients referred for AF ablation. In this cohort, HSAT demonstrated superior accuracy, compared to the STOP-BANG questionnaire and may be considered the preferred OSA screening tool in outpatient AF clinics.

**Graphical abstract:**

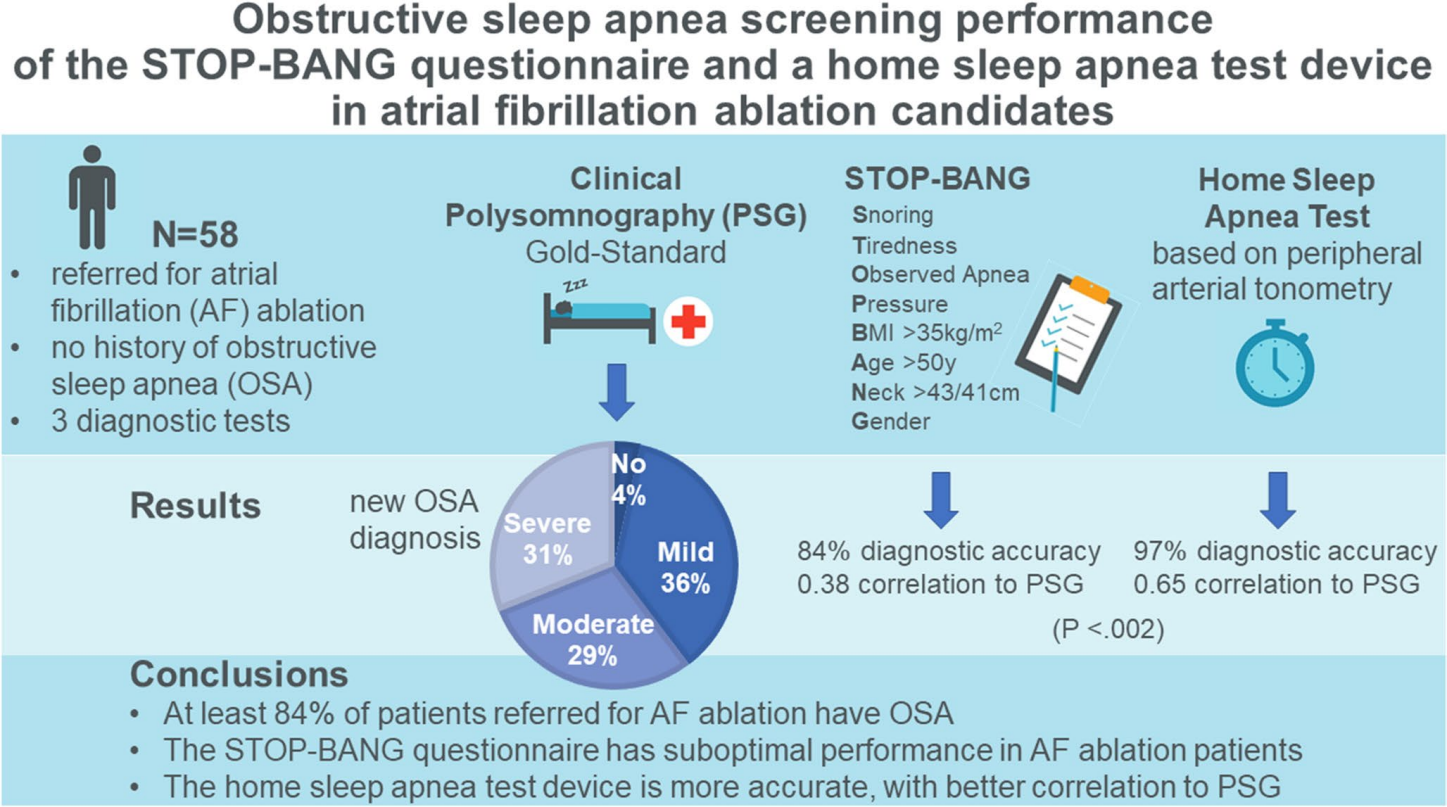

**Supplementary Information:**

The online version contains supplementary material available at 10.1007/s10840-025-02131-7.

## Highlights

OSA prevalence among AF ablation patients without a prior OSA diagnosis is 84%.

In AF ablation patients, the STOP-BANG questionnaire has suboptimal performance.

PAT-based HSAT identifies significantly more OSA than STOP-BANG in AF patients.

**Supplementary Information:**

The online version contains supplementary material available at 10.1007/s10840-025-02131-7.

## Background

Maintaining sinus rhythm in patients with atrial fibrillation (AF) remains challenging. In addition to pulmonary vein isolation (PVI) ablation, AF treatment is increasingly addressing pro-arrhythmic lifestyle risk factors, such as obesity and physical inactivity [1]. Evolving evidence suggests that effective rhythm control should also include the treatment of obstructive sleep apnea (OSA) [2]. OSA has various arrhythmogenic effects that contribute to the development of AF [3]. The prevalence of OSA in patients with AF is estimated to range from 21 to 74%, depending on the pattern and burden of AF [4]. Observational studies indicate that OSA treatment with continuous positive airway pressure (CPAP) reduces AF recurrence after PVI by 42% [2, 5]. Additionally, CPAP treatment induces electrical atrial reverse remodeling in AF patients [6]. Several combined lifestyle management studies that included OSA treatment with CPAP, reveal reductions in AF burden and improved freedom from AF [7–11]. This has led to the consensus that detecting and treating OSA could be considered for all patients eligible for AF PVI to improve rhythm control [12, 13].

The standard test for diagnosing OSA is polysomnography (PSG). While PSG provides comprehensive data on sleep respiratory disturbances, it is resource-intensive and not easily accessible for all patients. Consequently, initial screening for patients at risk for OSA might be conducted through more accessible tools, such as an OSA risk questionnaire [14]. Additionally, home sleep apnea tests (HSATs) using peripheral arterial tonometry (PAT) have emerged as cost-effective and less intrusive screening alternatives [15].

Despite the widespread availability of screening tools for OSA, their utility in AF clinics remains limited [16]. Daytime sleepiness, as assessed by the Epworth Sleepiness Scale (ESS), has shown limited reliability in predicting OSA [17, 18]. Additionally, the diagnostic accuracy of the STOP-BANG questionnaire in the AF population is uncertain, given that it was originally developed for preoperative screening [14]. This questionnaire emphasizes symptoms and physical features that are common in AF patients, which may compromise its specificity [18, 19]. Furthermore, a HSAT device using PAT might be susceptible to irregular heart rhythms and might potentially be a less reliable screening tool among AF patients. Therefore, particularly in the AF population requiring advanced rhythm control, the evaluation of these screening methods is essential.

This study aims to determine the screening value of the STOP-BANG questionnaire and a HSAT with PAT as a screening tool for OSA in AF patients referred for catheter ablation. By comparing both the performance of the STOP-BANG questionnaire and HSAT to a clinical PSG, this study aims to provide guidance on the most effective approach for identifying OSA in this high-risk population.

## Methods

### Study design and population

This prospective study was conducted at a tertiary referral hospital where PVI ablation is routinely performed. The current study’s cohort is a subsample from the randomized clinical Prevention to improve Outcomes of PVI (POP-AF) trial (Registration Number NCT05148338) [20]. The study was approved by the locally appointed medical ethics committee. Patients with symptomatic paroxysmal or persistent AF referred for their first PVI ablation and with at least one additional modifiable lifestyle risk factor (e.g. obesity, alcohol consumption, smoking) participated in the trial. Detailed inclusion and exclusion criteria were previously published in the study and design and rationale [21]. Patients randomized to the lifestyle intervention group consecutively underwent the STOP-BANG questionnaire and HSAT using PAT. Per study protocol, all patients with a PAT-derived apnea-hypopnea index (pAHI) of 5 or more per hour, based on a 3% oxygen desaturation threshold during HSAT, were referred to a sleep clinic for a clinical VideoPSG. For the current substudy, we included all patients who underwent the clinical PSG. Patients that were already diagnosed for OSA, or that refused clinical PSG were excluded.

### Administration of the STOP-BANG questionnaire

The STOP-BANG questionnaire was administered by a specialized nurse-practitioner, who also measured body weight and neck circumference. The STOP-BANG is a scale scoring from 0 to 8, depending on the presence of different parameters: Snoring (S), Tiredness (T), Observed apnea episodes (O), high blood Pressure (P), Body Mass Index (BMI) > 35 kg/m^2^ (B), Age > 50 years old (A), Neck circumference ≥ 43 cm in men and ≥ 41 cm in women (N) and male Gender (G).

### Home sleep apnea test

All participants underwent one night at home with the HSAT device WatchPAT 300 (Zoll Itamar Medical Europe, Amsterdam, the Netherlands). This device captures posture, snoring, and chest motion using a chest sensor; oxygen saturation, heart rate and peripheral arterial tonometry at the fingertip as well as actigraphy at the wrist. The algorithm deduces sleep state, sleep stages, and respiratory events. The device has previously been validated in AF patients and is able to estimate OSA severity [22]. Beforehand, patients were instructed by the specialized-nurse practitioner on how to apply the test at home. All recordings were analyzed with the zzzPAT software (version 5.2.79.7) and manually reviewed to verify the quality of the data.

### Clinical videopolysomnography

The clinical PSG was performed according to the American Academy of Sleep Medicine (AASM) standards, including electroencephalography (EEG), electromyography, and electro-oculography to score sleep, full respiratory recordings including oronasal thermistor and flow, thoracic abdominal respiratory inductance plethysmography, oxygen saturation and lead II electrocardiogram, as described elsewhere [23]. All recordings were scored according to the AASM standards with respect to sleep staging and respiratory events [24]. Accordingly, an apnea was scored if flow peak signal dropped by 90% for at least 10 s. A hypopnea was scored for any 10-second reduction in airflow by 30%, accompanied by a 3% desaturation or an arousal. The apnea-hypopnea index (AHI) was calculated as the total number of apneas plus hypopneas divided by the total sleep time. Patients were diagnosed with OSA if they had an AHI of 5 per hour or higher. Based on AASM OSA severity classification, patients were defined to have mild OSA (AHI ≥ 5 and < 15 events per hour), moderate OSA (AHI ≥ 15 and < 30 events per hour) or severe OSA (AHI ≥ 30 events per hour).

### Statistical analysis

The patient characteristics are presented as absolute numbers and percentages for categorical variables and as mean with standard deviation (SD) or median with interquartile range (IQR) for continuous variables depending on normality. Differences in categorical variables between the clinical OSA severity groups are tested using the Chi-square test or Fisher’s exact test as appropriate. For continuous variables, the analysis of variance is used to compare groups and for non-normally distributed variables the Kruskal-Wallis test is used. The diagnostic values for OSA screening of the STOP-BANG questionnaire and of the HSAT are calculated, compared to the PSG. McNemar mid-*P* test is used to compare the accuracy of tests. The correlation between the STOP-BANG or HSAT and PSG were tested with Pearson or Spearman test as appropriate. A *P*-value < 0.05 is considered statistically significant. Statistical analyses are performed with R statistics 4.2.1 (R Foundation for Statistical Computing).

## Results

### Patient inclusion

**Fig. 1:**
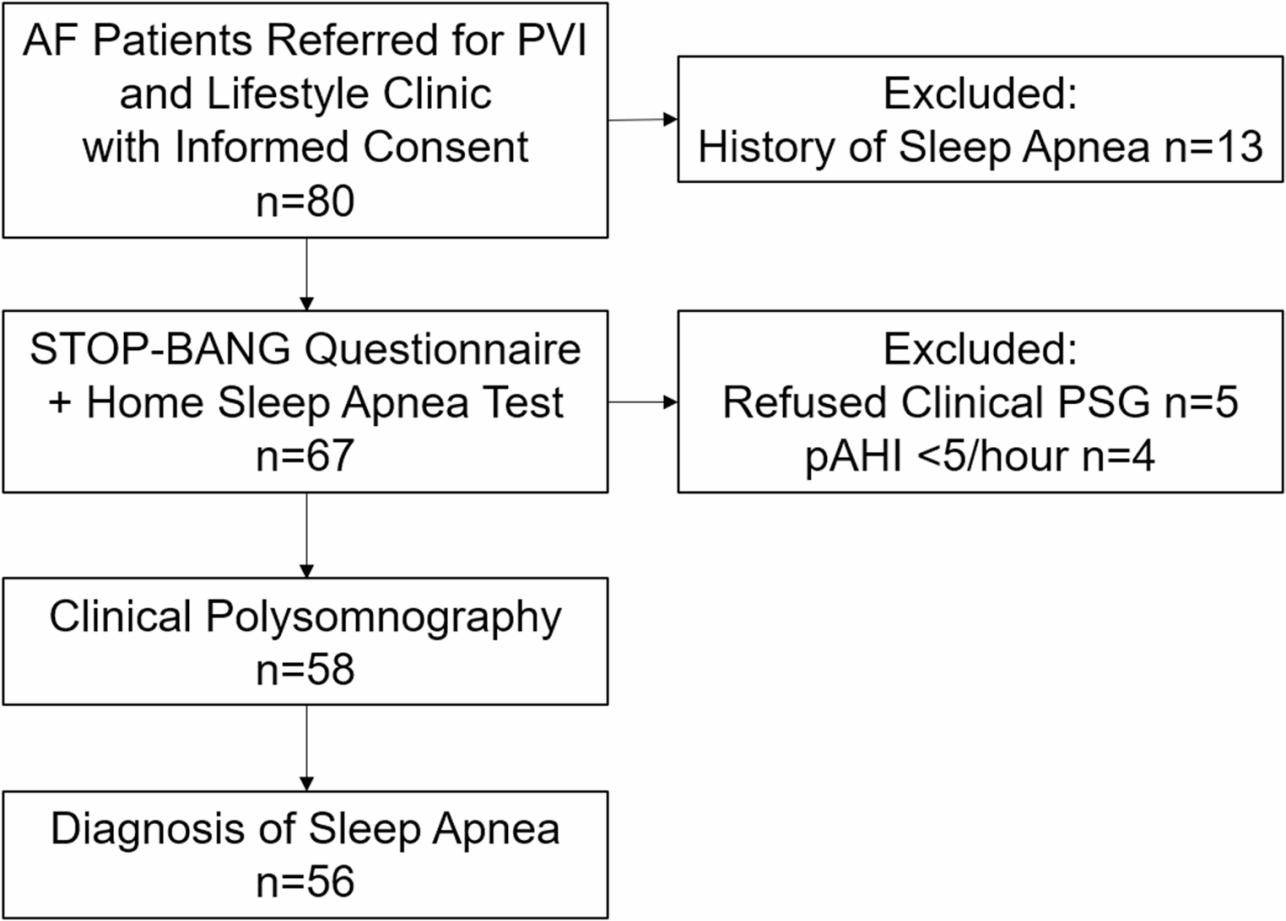
Patient Study Flow Chart. AF, atrial fibrillation; pAHI, peripheral arterial tonometry-derived apnea-hypopnea index; PSG, polysomnography; PVI, pulmonary vein isolation

After excluding patients with a history of OSA, a total of 67 patients underwent both the STOP-BANG questionnaire and HSAT device. Among these, 5 patients declined to further undergo clinical PSG and 4 patients had a pAHI below 5 per hour on HSAT. Consequently, the final analysis included 58 patients who fully completed the STOP-BANG questionnaire, the ambulatory sleep test and the PSG (Fig. 1). Among patients undergoing the PSG, the median age was 62 years and 38% were female. In this cohort, most patients were overweight, with 97% having a BMI over 25 kg/m^2^ and 48% classified as obese, while 50% had hypertension. Persistent AF was present in 57% of patients; 93% had a good (> 50%) left ventricular ejection fraction and median NT-proBNP was 170 pg/mL.

### Diagnosis of OSA and OSA severity

Among the 67 patients without a prior medical history of sleep apnea, 56 (84%) were found to have OSA. Clinical PSG confirmed the diagnosis in 56 out of 58 (97%) patients that underwent PSG. Among those patients, OSA was classified in 38% as mild, in 30% as moderate, and in 32% as severe. Age and BMI were statistically different between these OSA severity clusters. An increase in age was associated with an increase in OSA severity (*P*-value 0.016). In those with any OSA, a higher BMI was associated with an increase in OSA severity (*P*-value 0.024). Lifestyle characteristics such as hypertension, hypercholesterolemia, diabetes mellitus and alcohol consumption did not differ significantly between OSA groups. The ESS (median 7) did also not differ significantly between groups. The OSA severity groups were rather small, so caution should be used when interpretating OSA group differences. The baseline demographics are displayed in Table 1.

**Table 1:** Baseline demographics

PSG diagnosis	Total	No OSA (AHI < 5/h)	Mild OSA (AHI 5–15/h)	Moderate OSA (AHI 15–30/h)	Severe OSA (AHI ≥ 30/h)	*P*-value
Total (N)	58	2	21	17	18	
Female Sex	22 (38%)	0 (0%)	9 (43%)	6 (35%)	7 (39%)	0.82
Age (year)	62 [57;67]	45 [37;52]	59 [56;63]	62 [59;65]	67 [63;69]	**0.016**
BMI (kg/m^2^)	30.0 [28.2;33.0]	34.7 [32.3;37.1]	28.8 [27.5;31.6]	29.4 [28.6;30.3]	32.2 [30.2;34.0]	**0.024**
Hypertension	29 (50%)	1 (50%)	6 (29%)	11 (65%)	11 (61%)	0.08
Hypercholesterolemia	20 (35%)	0 (0%)	6 (29%)	7 (41%)	7 (39%)	0.70
Diabetes mellitus	4 (6.9%)	0 (0%)	1 (4.8%)	2 (12%)	1 (5.6%)	0.72
Alcohol consumption (> 6 units per week)	15 (26%)	1 (50%)	2 (9.5%)	5 (29%)	7 (39%)	0.12
AF Pattern						0.33
-Paroxysmal	25 (43%)	1 (50%)	6 (29%)	8 (47%)	10 (56%)	
-Persistent	33 (57%)	1 (50%)	15 (71%)	9 (53%)	8 (44%)	
CHA_2_DS_2_-VASc	2 [1;2.75]	1 [0.5;1.5]	1 [1;2]	1 [1;3]	2 [2;3]	0.15
LVEF						0.17
-good (> 50%)	54 (93%)	2 (100%)	19 (91%)	16 (94%)	17 (94%)	
-moderate (30–50%)	4 (6.9%)	0 (0%)	2 (9.5%)	1 (5.9%)	1 (5.6%)	
NT-proBNP (pg/mL)	170 [61;392]	186 [116;256]	227 [41;448]	101 [59;168]	188 [140;362]	0.46
ESS	7.0 [3.5;9.5]	4.0 [3.5;4.5]	6.0 [2.0;9.0]	7.5 [5.0;8.8]	8.0 [3.0;10.5]	0.55

AHI, apnea-hypopnea index; BMI, body mass index; CHA_2_DS_2_-VASc, congestive heart failure, hypertension, age ≥ 75 (doubled), diabetes, stroke (doubled), vascular disease, age 65–74, sex category (female); ESS; Epworth Sleepiness Scale; LVEF, left ventricular ejection fraction; NT-proBNP, N-terminal pro B-type natriuretic peptide; OSA, obstructive sleep apnea; PSG, polysomnography

All 56 patients diagnosed with sleep apnea were found to have obstructive sleep apnea. In addition, 7 of these patients also exhibited central apnea present, leading to a diagnosis of mixed apnea. The total sleep time during clinical PSG averaged 377 min and did not differ significantly between OSA severity groups (*P*-value 0.22). However, the oxygen desaturation index for 4% desaturation differed significantly across OSA severity classes (*P*-value < 0.001), as did the duration of oxygen saturation under 90% (*P*-value < 0.001). Notably, 10 patients experienced paroxysms of AF during PSG. Further polysomnography outcomes are summarized in Table 2.

**Table 2:** Polysomnography outcomes

PSG diagnosis	Total	No OSA (AHI < 5/h)	Mild OSA (AHI 5–15/h)	Moderate OSA (AHI 15–30/h)	Severe OSA (AHI ≥ 30/h)	*P*-value
Total (N)	58	2	21	17	18	
Additional mixed apnea diagnosis	7 (12.1%)	0 (0.0%)	1 (4.8%)	1 (5.9%)	5 (27.7%)	0.11
Total sleep time (mins)	377 (75.4)	393 (39.6)	403 (70.7)	367 (85.1)	355 (69.3)	0.22
AHI3 (%)	17.2 [11.0;35.7]	1.50 [1.25;1.75]	10.0 [6.80;12.8]	20.0 [16.7;23.7]	44.3 [38.0;52.2]	< 0.001
ODI4 (%)	4.40 [2.12;11.9]	1.05 [0.68;1.43]	2.30 [1.60;4.10]	4.40 [3.40;7.40]	24.6 [13.0;33.6]	< 0.001
O2Sat ≤ 90% (mins)	7.0 [1.5;22.0]	0.0 [0.0;0.0]	2.6 [0.9;11.9]	2.0 [0.6;6.5]	24.2 [13.4;38.2]	0.001
Atrial fibrillation during PSG	10 (17.2%)	0 (0.0%)	4 (19.0%)	1 (5.9%)	5 (27.8%)	0.36

AHI3, apnea-hypopnea index for 3% desaturation; ODI4, oxygen desaturation index for 4% desaturation; OSA, obstructive sleep apnea; O2Sat, oxygen saturation; PSG, polysomnography

### Results of the STOP-BANG questionnaire

**Fig. 2:**
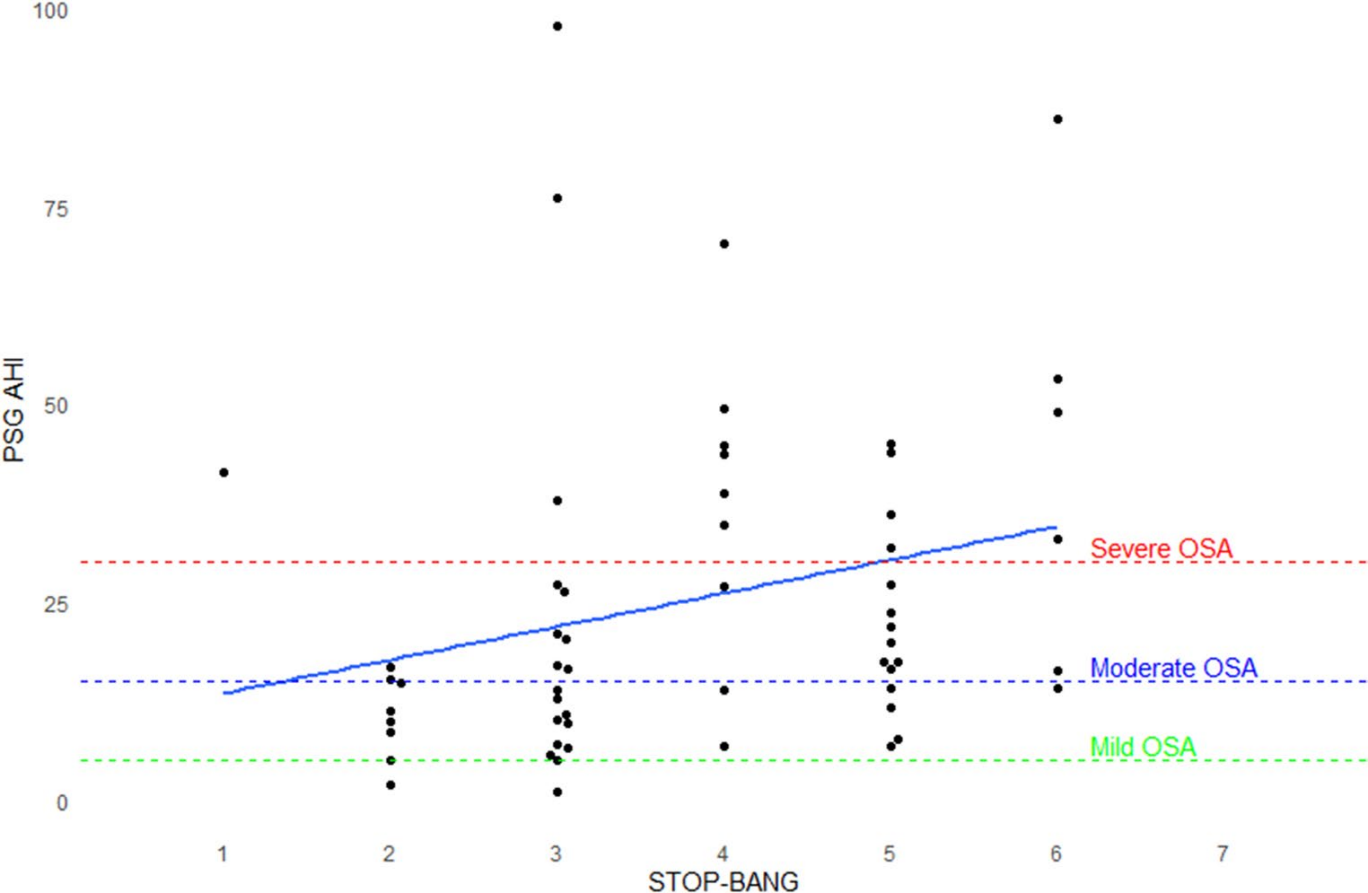
Strip Chart and correlation of STOP-BANG scores and AHI on PSG. AHI, apnea-hypopnea index; OSA, obstructive sleep apnea; PSG, polysomnography

Among the studied patients, the median STOP-BANG score was 4, with a score range from 1 to 6. A STOP-BANG score ≥ 3 was present in 84% of patients (see Supplementary materials Table S1). The STOP-BANG score correlated poorly with AHI on PSG (Spearman’s Rho 0.38, *P*-value = 0.003). See Fig. 2.

The usual cut-off score of ≥ 3 had a sensitivity of 86% and specificity of 50%, and accuracy of 84% for any form of OSA. Sensitivity of the STOP-BANG was higher for detecting moderate and severe OSA, or severe OSA, when compared to all forms of OSA. (See Table 3) The accuracy of the STOP-BANG was significantly different from the clinical PSG (*P*-value < 0.021).

**Table 3:** The sensitivity and specificity for different STOP-BANG cut-off scores

STOP-BANG Cut-off	2 or higher	3 or higher	4 or higher	5 or higher	6 or higher
Any OSA (AHI 5/h or higher)					
Sensitivity	98%	86%	54%	38%	11%
Specificity	0%	50%	100%	100%	100%
Moderate or Severe OSA (AHI 15/h or higher)					
Sensitivity	97%	91%	66%	46%	14%
Specificity	0%	26%	70%	78%	96%
Severe OSA (AHI 30/h or higher)					
Sensitivity	94%	94%	78%	44%	22%
Specificity	0%	20%	60%	68%	95%

AHI, apnea-hypopnea index; OSA, obstructive sleep apnea

### Results of the home sleep apnea test

**Fig. 3:**
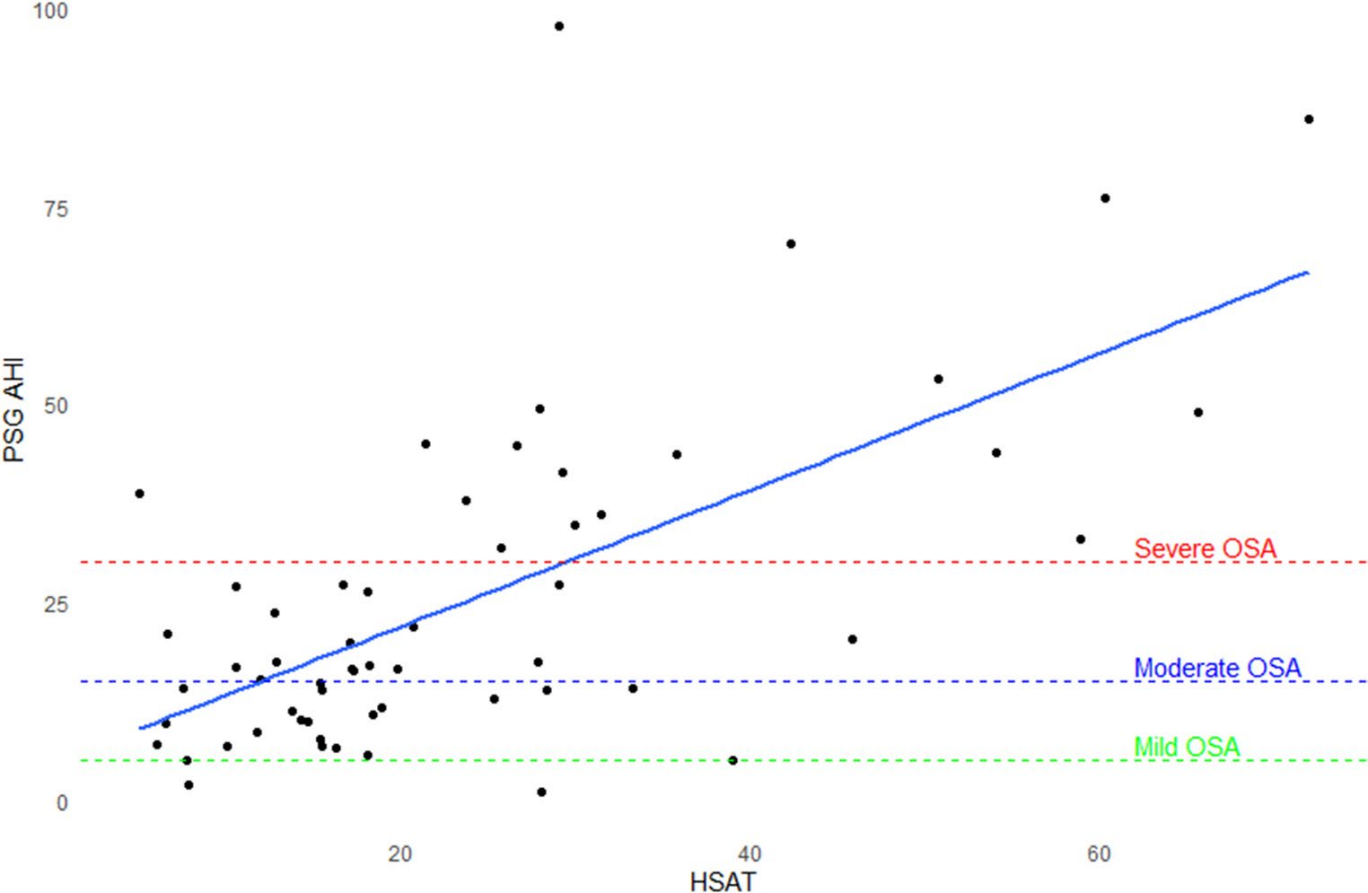
Scatterplot and correlation for HSAT AHI versus PSG AHI. AHI, apnea-hypopnea index; HSAT, home sleep apnea test; OSA, obstructive sleep apnea; PSG, polysomnography

In the study cohort of patients referred for clinical PSG, the median pAHI from the HSAT was 18.3/hour. An AHI of ≥ 15/hour was present in 60% of patients. The mean sleeping time with HSAT was 399 min. A total of 9 patients experienced AF during their HSAT study. Further HSAT outcomes are displayed in Supplementary material Table S2. The pAHI on HSAT correlated moderately with the AHI on PSG (Pearson’s coefficient 0.65, P-value < 0.001), as shown in Fig. 3.

The sensitivity and specificity for the HSAT pAHI cut-off scores 15/hour and 30/hour were determined for respectively all forms of OSA, moderate or severe OSA, and for severe OSA. Sensitivity of the HSAT was higher for detecting moderate and severe OSA, or severe OSA, when compared to all forms of OSA. (See Table 4). Following the study protocol, there were no patients with pAHI below 5/hour referred for clinical PSG, so the cut-off score of 5/hour was not available. The accuracy of screening with HSAT was similar to the clinical PSG (*P*-value = 0.25). Notably, the performance of the HSAT was significantly more accurate than the STOP-BANG questionnaire (*P*-value < 0.002).

**Table 4:** The sensitivity and specificity for pAHI 15 and pAHI 30 on HSAT

HSAT pAHI Cut-off	15 per hour or above	30 per hour or above
Any OSA (AHI 5/h or higher)		
Sensitivity	71%	23%
Specificity	50%	100%
Moderate or Severe OSA (AHI 15/h or higher)		
Sensitivity	80%	31%
Specificity	43%	91%
Severe OSA (AHI 30/h or higher)		
Sensitivity	94%	56%
Specificity	40%	93%

AHI, apnea-hypopnea index; HSAT, home sleep apnea test; OSA, obstructive sleep apnea; pAHI, peripheral arterial tonometry-derived apnea-hypopnea index; PSG polysomnography

## Discussion

The main findings of our study are that (1) OSA is highly prevalent in patients referred for AF ablation; (2) age and BMI are independent predictors for OSA severity based on AHI; (3) daytime sleepiness is low among AF patients and does not correlate to OSA severity based on AHI; (4) the STOP-BANG questionnaire is a suboptimal screening instrument in this high-risk AF population; (5) patients with AF and a high risk of OSA can be adequately screened with a HSAT device based on PAT.

### Prevalence of OSA in unscreened AF patients

In this study, we evaluated a cohort of symptomatic AF patients referred to a tertiary hospital for PVI ablation, all of whom had at least one additional modifiable lifestyle risk factor. As anticipated, nearly all patients in this cohort were overweight or obese. Other modifiable risk factors included hypertension and excessive alcohol consumption. Our cohort demonstrated low daytime sleepiness scores, which was comparable to previous observations in patients with AF [17, 18]. Notably, among all patients referred for AF ablation without previous OSA diagnosis, 84% demonstrated to have at least a mild form of OSA, and 52% had at least moderate OSA based on clinical PSG. Previously, a comparable prevalence was reported in patients referred for ablation [25–27]. Although the generalizability to all AF patients is limited, the high prevalence of sleep apnea highlights the importance of OSA screening in this selected population.

### Screening value of STOP-BANG questionnaire

In our study, a STOP-BANG cut-off score of 3 showed high sensitivity but low specificity in this high-risk group. We revealed that a STOP-BANG score of 3 or more was 86% sensitive and 50% specific when compared to a clinical PSG. Given that almost all patients were overweight and had a high burden of AF, the low specificity of the STOP-BANG questionnaire was expected, as many of the questionnaire’s features overlap with risk factors for AF. The diagnostic values of our study are consistent with previous findings in AF patients when compared to ambulatory PSG [18, 28]. In order to improve the diagnostic performance for intermediate OSA risk patients with new-onset AF, the addition of four clinical questions to the STOP-BANG was proposed, indicating the low performance of the STOP-BANG [29].

### Screening value of watchpat device

The HSAT demonstrated a good performance in screening this symptomatic AF population, accurately identifying OSA patients that were missed with the STOP-BANG questionnaire. The pAHI on HSAT correlated well with the AHI as determined with the gold standard clinical PSG. However, due to the design of this study, 4 patients with a low pAHI on HSAT (< 5/hour) did not receive a clinical PSG and hence their OSA status according to the gold standard remains unknown, limiting our ability to determine sensitivity and specificity. Therefore, we cannot state whether a pAHI lower than 5 per hour excludes OSA, and determine the role of clinical PSG in these low-risk patients.

The additional diagnostic yield of conducting a clinical PSG in patients with a positive HSAT was limited in our cohort, as the positive predictive value of pAHI of more than 5 per hour for any form of OSA was 98%. Only two patients were not diagnosed with OSA, while seven were reclassified from mild to moderate (*n* = 6) or mild to severe (*n* = 1) OSA, possibly impacting the urgency of their OSA treatment. Interestingly, night-to-night variability of OSA in relation to AF might be a an explanation for this incoherence [30]. Nevertheless, in line with our research, the WatchPAT device was validated as a reliable screening tool among AF patients and able to estimate OSA severity [22]. An upcoming clinical trial will be evaluating HSAT in ambulatory patients with AF to give further practical guidance on the use of HSAT [27]. However, the study will not include PSG as gold standard test.

### Strengths and limitations

Our study’s strengths include the use of clinical PSG as the gold standard test for diagnosing OSA. However, due to the study design as part of a prospective clinical trial, patients with a low pAHI on HSAT (lower than 5 per hour) did not receive a clinical PSG. We do not expect these 4 patients with pAHI of < 5/hour to have sleep apnea, given that HSAT tends to measure higher AHI values than PSG [31].

A limitation of our study is the sparse data on patients without OSA. Additionally, HSAT and PSG were not performed simultaneously, which may lead to different results. Furthermore, AHI determination from HSAT might be prone to night-to-night variation [31]. Lastly, the total sleep time, a key factor in calculating AHI, is determined more accurately by EEG in PSG than in HSAT. However, in our study, the mean total sleep time was comparable between HSAT (399 min) and PSG (377 min).

As this study is performed in patients who are referred for PVI ablation with an additional lifestyle risk factor, the generalizability to all AF patients is limited. The group of patients referred for PVI might be less susceptible to anti-arrhythmic drug therapy, i.e. more difficult to treat. The high prevalence of underlying untreated OSA might be associated with this treatment difficulty, as diminished response to drug therapy was demonstrated in patients with OSA [32].

### How to implement OSA screening in the AF clinic?

Currently, only 11.3% of AF clinics systematically test for OSA, while the majority (67.7%) conduct OSA testing on an ad-hoc basis, primarily due to lack of an established collaboration between AF clinics and sleep clinics [16]. The OSA prevalence in our study supports the need for more standard OSA screening. While the STOP-BANG questionnaire is freely available, easy to administer, and requires no equipment, its lower diagnostic accuracy in this cohort limits its utility. In contrast, HSAT offers significantly higher accuracy but may involve additional logistical complexity and costs. These factors should be considered when selecting a screening strategy, particularly in resource-limited settings or where cost-effectiveness is a priority. In most cases (97%), referral to a sleep clinic following HSAT will not alter the diagnosis. However, establishing a robust collaboration between sleep clinics and cardiology departments is essential for improving diagnosis and treatment of OSA.

It remains a topic of debate whether OSA with an AHI < 15/hour, without OSA-related symptoms, should be treated and if such treatment improves AF symptoms and morbidity. In OSA patients with paroxysmal AF, CPAP did not reduce the risk of AF recurrence following ablation, though the study reporting these findings may have been underpowered [33]. Similarly, CPAP did not improve AF recurrence after cardioversion in patients with AHI > 5/hour [5]. Conversely, several studies on integrated lifestyle modifications, including OSA treatment, have revealed favorable AF outcomes, supporting the justification for detecting and treating OSA in patients eligible for AF PVI [7–11]. In addition to AHI, other important treatment indicators related to cardiovascular disease might include desaturation depth and duration. In our study, however, the oxygen desaturation index for 4% desaturation and the duration of oxygen saturation below 90% were specifically associated with OSA severity as defined by AHI. A study on deeper understanding of OSA pathophysiology in AF patients is underway (NCT06246825) and might benefit patient-tailored treatments and further improve outcomes.

## Conclusion

We determined the screening performance for OSA of the STOP-BANG questionnaire and WatchPAT device in a population of symptomatic AF patients presenting at a tertiary AF clinic, using a clinical PSG as gold standard test. Among 67 OSA naïve patients referred for their first AF ablation whom had an additional lifestyle risk factor, 56 (84%) were demonstrated to have at least mild OSA. The high prevalence of OSA in this cohort underscores the importance of screening for OSA in AF ablation patients. In this AF population, the STOP-BANG questionnaire was a less accurate screening tool with suboptimal sensitivity and specificity, compared to the HSAT, when using PSG as the standard-reference, and hence HSAT is well suited for OSA screening in the outpatient AF clinic. The design of the study limits any conclusions on patients with a negative HSAT (pAHI lower than 5 per hour). Further research is needed to refine these screening methods and determine their impact on clinical outcomes in AF patients.

## Supplementary Information

Below is the link to the electronic supplementary material.

Supplementary Material 1 (DOCX 91.0 KB)

## Data Availability

Data from the POP-AF trial are available upon reasonable request to the first author, sub-investigator Jasper Vermeer. He had full access to all the data and takes responsibility for its integrity and data analysis. The authors are solely responsible for the design and conduct of this study, all study analyses, the drafting and editing of the paper and its final contents.
