# Supplementary figures and images for: Obstructive sleep apnea screening performance of the STOP-BANG questionnaire and a home sleep apnea test device in atrial fibrillation ablation candidates
Source: J Interv Card Electrophysiol. 2025 Oct 18;69(2):239–48. doi: 10.1007/s10840-025-02131-7 (PMC12876079; doi:10.1007/s10840-025-02131-7)

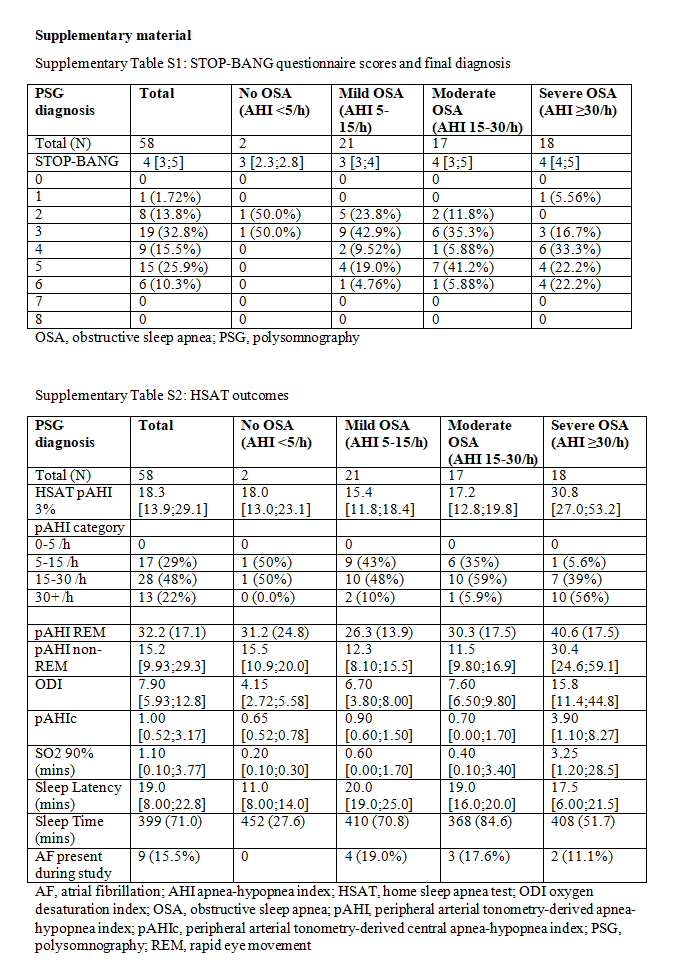

Supplement: Supplementary file 1 — Supplementary Material 1 (DOCX 91.0 KB) [file 10840_2025_2131_MOESM1_ESM.docx]
